# Robot-assisted CT-guided cryoablation of pulmonary metastases: an IDEAL stage 2a prospective development study

**DOI:** 10.1007/s00330-026-12335-8

**Published:** 2026-02-05

**Authors:** Nicos Fotiadis, Sajjan KC, Shaira Farooq, Jodie Basso, David Cunningham, Dow-Mu Koh, S. Nahum Goldberg, Edward W. Johnston

**Affiliations:** 1 Interventional Radiology, Royal Marsden Hospital, London, UK; 2 Radiotherapy and Imaging, Institute of Cancer Research, Sutton, UK; 3 Gastrointestinal Oncology Unit, The Royal Marsden Hospital, London, UK; 4 Medical Oncology, Institute of Cancer Research, Sutton, UK; 5 Diagnostic Radiology, Royal Marsden Hospital, London, UK; 6 Hadassah Hebrew University Medical Center, Jerusalem, Israel; 7 Beth Israel Deaconess Medical Center, Harvard Medical School, Boston, MA USA

**Keywords:** Robotic guidance, CT-guided cryoablation, Lung metastases, Prospective development study, Interventional oncology

## Abstract

**Objectives:**

To evaluate the feasibility, safety, and technical performance of robot-assisted CT-guided cryoablation for pulmonary metastases.

**Materials and methods:**

A single-centre IDEAL stage 2a prospective development study of 26 participants (median age 62 years, IQR 47–71; 14 men) who underwent 30 procedures targeting 37 lung metastases using a robotic navigation system. Median tumour diameter was 9.8 mm (IQR 5.1–12.8). All procedures were performed under general anaesthesia with high-frequency jet ventilation. Feasibility, safety, and technical performance (targeting accuracy, manipulations, radiation dose) were recorded.

**Results:**

Robotic guidance was successfully completed without conversion in 35/37 tumours (95%). One major complication occurred (3%, CTCAE grade 3 pneumothorax requiring 4 days of drainage); all others were grade 1–2. Pneumothoraces were managed by observation (*n* = 7) or prophylactic intraprocedural chest drain insertion (*n* = 11). No bronchopleural fistulas were observed. Median hospital stay was 1 night (IQR 1–2). A total of 54 cryoprobes were used. Median Euclidean targeting error on first insertion was 6.1 mm (IQR 2.9–9.7) and lateral error 4.2 mm (IQR 2.2–6.5). The median number of manipulations per probe was 1 (IQR 0–2.5), with one-third requiring no adjustment. Once integrated into the workflow, the “chopstick” technique was frequently applied, supporting conformal ablation. Median total procedure time was 66.5 min (IQR 56.6–92.8). Twelve-month local tumour progression-free survival was 97%.

**Conclusion:**

Robot-assisted CT-guided cryoablation of pulmonary metastases was feasible, safe, and accurate, achieving high targeting precision with minimal cryoprobe manipulation. These findings support evaluation in prospective comparative trials.

**Key Points:**

***Question*** *Robotic-assisted CT-guided cryoablation of lung metastases is feasible and safe, achieving high targeting accuracy and minimal probe manipulation, even in anatomically challenging cases.*

***Findings*** *Robotic trajectory planning supported complex multiprobe configurations. Procedural refinements—including patient positioning, probe selection, and adoption of “chopstick” configurations—were introduced to address bleeding risk and optimise energy delivery.*

***Clinical relevance*** *Robot-assisted navigation is particularly advantageous in cryoablation, enabling minimal manipulations and accurate probe placement despite the often-necessary complex trajectories.*

**Graphical Abstract:**

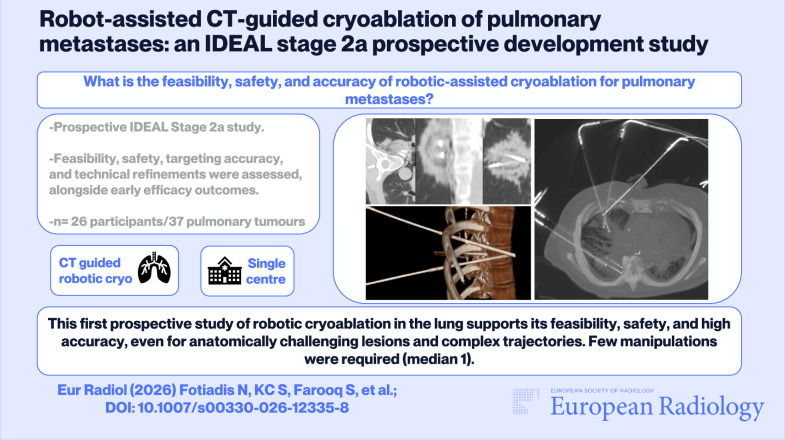

## Introduction

Percutaneous cryoablation is a well-established, locally curative treatment that provides durable local control for pulmonary metastases [1, 2]. It is particularly advantageous for subpleural tumours due to its collagen-sparing mechanism, which lowers the risk of bronchopleural fistula compared to heat-based ablation techniques [3].

However, cryoablation presents distinct technical challenges. Precise probe placement is essential and often demands complex trajectories to avoid fissures and navigate between ribs. These difficulties are amplified by the “chopstick technique”, which refers to the placement of two cryoprobes that flank the tumour and do not touch each other, akin to chopsticks, to tailor probe spacing and achieve a larger, confluent ablation zone [4]. Freehand placement usually requires multiple iterative adjustments to obtain optimal needle configuration, increasing procedure time and radiation dose, and potentially the risk of haemorrhage or pneumothorax, with reported pneumothorax rates exceeding 60% [5].

Stereotactic and robotic navigation platforms have been shown to improve targeting accuracy and reduce needle adjustments in liver ablation [6–8]. Although evidence in lung ablation is limited, early feasibility of robot-assisted radiofrequency ablation has demonstrated reduced need for probe manipulation with an encouraging safety profile [9]. However, their application to cryoablation, where multiprobe precision is critical to achieving overlapping isotherms, remains uncertain.

This study was designed within the framework of the IDEAL recommendations for surgical and interventional innovation [10]. IDEAL provides a staged model for evaluating new techniques, ranging from first-in-human studies (Stage 1) through to multicentre randomised trials (Stage 4). This work corresponds to IDEAL Stage 2a, in which iterative refinement, workflow optimisation, and reproducibility are emphasised before progression to comparative multicentre evaluation. We evaluate the feasibility, safety, and technical performance of robotic-assisted CT-guided cryoablation for subpleural lung metastases, documenting iterative refinements during early implementation.

## Materials and methods

### Study design and oversight

This was a single-centre prospective development study approved by the institutional review board (identifier 1062), where all participants provided informed consent.

### Eligibility

Eligible participants were adults with one or more pulmonary metastases referred for image-guided thermal ablation with curative intent. Inclusion criteria were: (1) one or more metastases amenable to percutaneous ablation; (2) subpleural or central location of lesion(s) favouring cryoablation; and (3) multidisciplinary tumour board consensus of appropriateness of the treatment strategy. Exclusion criteria were: (1) procedures performed using radiofrequency ablation; (2) unsuitable for general anaesthesia; and (3) active infection.

### Procedural technique

**Fig. 1:**
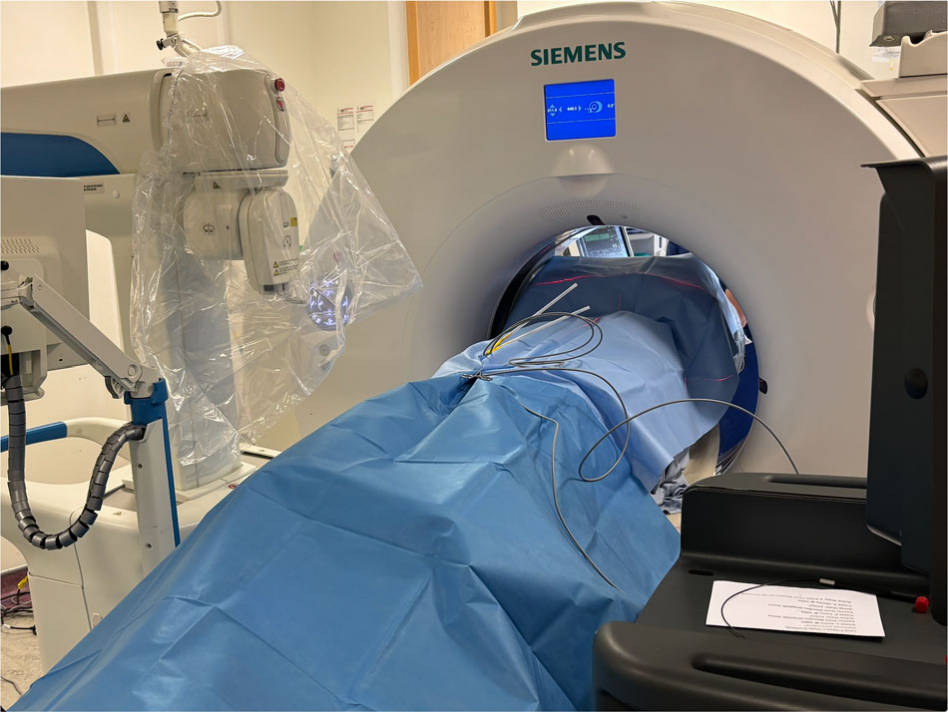
Procedural setup. Procedural setup for robot-assisted CT-guided lung cryoablation showing the robotic arm (left, plastic-covered), CT gantry, and immobilised, draped patient under general anaesthesia with high-frequency jet ventilation. Cryoprobes and cabling are also visible

All procedures were performed under general anaesthesia with high-frequency jet ventilation (Monsoon Acutronic Jet Ventilation System III) to minimise respiratory motion. The robotic navigation system is a CE-marked, FDA-approved (Maxio, Perfint Healthcare) device operated within our 64-slice interventional CT suite (Definition Edge, Siemens Healthineers) [11]. Vacuum mattress immobilisation (Klarity Vacuum Bag) was used to minimise external patient motion. The robotic system was docked during routine sterile preparation and cleaning of the patient before the planning CT scan. Setup from a typical case is provided in Fig. 1. Docking typically required approximately 5 min, performed in parallel with patient preparation, and therefore did not add materially to procedure time. Volumetric, unenhanced CT imaging was acquired (3 mm slice thickness, 1 mm interval) for planning, confirmation, monitoring and final imaging.

Target tumours were segmented on the MAXIO workstation using its semi-automatic contouring tool, and trajectories were planned using illustrated isotherms to ensure complete target coverage and avoid critical structures. One or two cryoprobes (14 G IceForce, 17 G IceRod or IceSphere, Boston Scientific) were planned per target tumour. The robotic system permits both orbital and craniocaudal angulation. Where possible, for simplicity, we preferentially selected an in-plane axial route, which resulted in a median craniocaudal angulation of 0°. Ablation zone planning used the manufacturer-provided isotherm models within the robotic system to guide probe positioning and anticipated coverage. Intra-procedurally, coverage was assessed visually on CT based on the extent of ground-glass opacity relative to the tumour, rather than from software-derived estimates. This visual approach is well-suited to lung cryoablation, where both the tumour and probes are directly visible on CT, allowing confident assessment of probe–tumour and corresponding ablation zone relationships without image fusion.

The robotic arm docks externally to a fixed floor plate adjacent to the CT table. It remains outside the gantry bore, allowing unrestricted gantry travel. The electromechanical arm executed planned trajectories in physical space. A laser marked the entry point, guiding a skin incision through which cryoprobes were advanced via the needle guide. Cryoprobe advancement is performed manually outside the gantry in a single step along the planned trajectory using the robotic guidance system, which provides high positional accuracy and obviates the need for incremental imaging. Pleural anaesthesia was not used, since even minor pneumothoraces can cause target displacement and loss of registration accuracy; therefore, additional pleural punctures were deliberately avoided to minimise this risk. Final positioning was confirmed with CT once all probes were positioned. Manual adjustments were made where necessary, and each manipulation was recorded. Coaxial needles were not used.

**Fig. 2:**
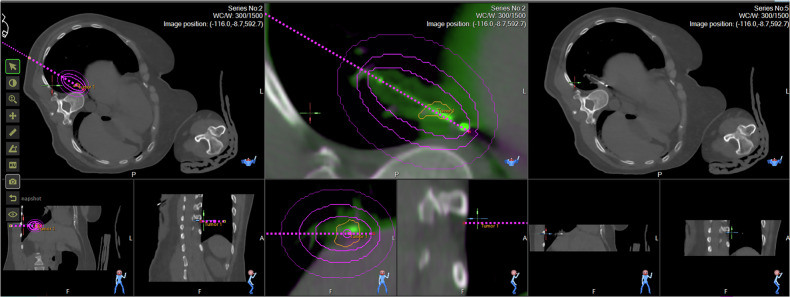
Single-cryoprobe plan and targeting accuracy. Robotic planning and verification display for a 76-year-old man undergoing cryoablation of a subpleural right lower-lobe colorectal metastasis. Left: Planning CT showing the intended cryoprobe trajectory with manufacturer-supplied isotherms. Centre: Fused overlay of planning and verification CT demonstrating close alignment between planned (magenta) and actual (green) needle paths. Right: Verification CT confirming accurate single-probe placement

**Fig. 3:**
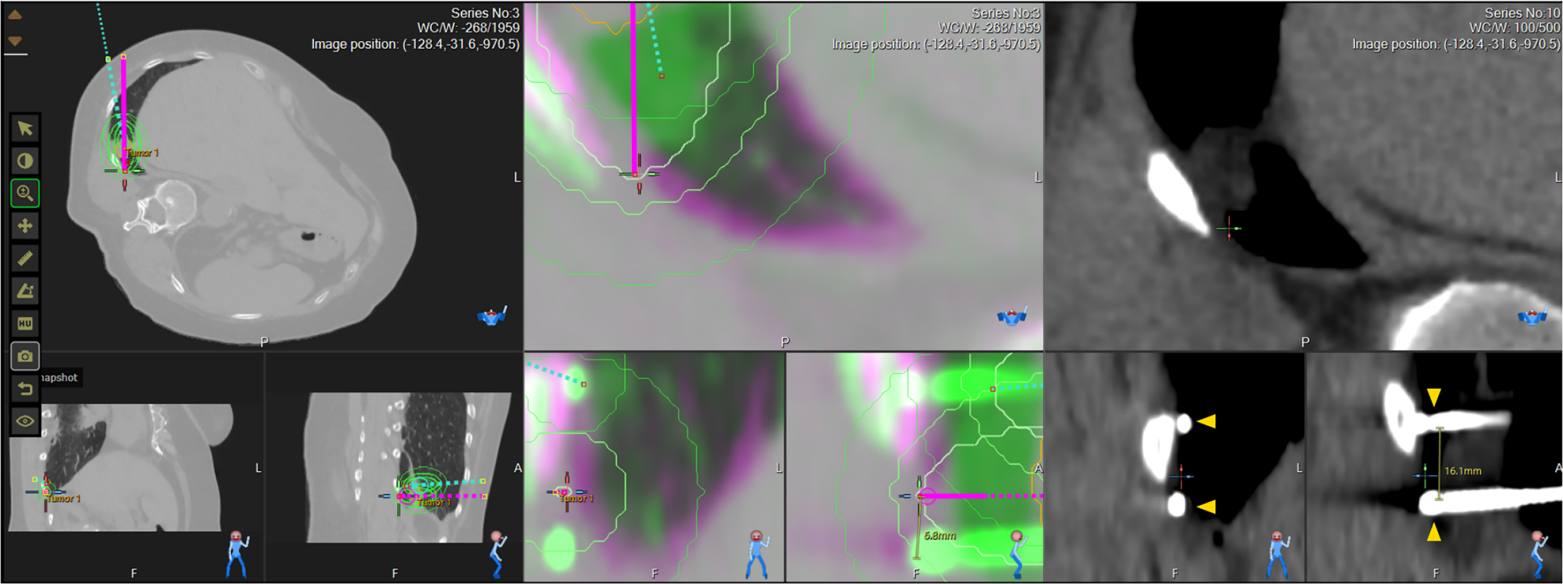
‘Chopstick’ plan and targeting accuracy. A 63-year-old woman undergoing cryoablation of a subpleural right lower-lobe colorectal metastasis. Left: Planning CT showing intended cryoprobe trajectories and manufacturer-supplied isotherms. Centre: Fused planning and verification CT demonstrating assessment and measurement of targeting accuracy between planned (magenta) and actual (green) needle paths. Right: Verification CT showing final cryoprobe positions in a “chopstick” configuration (yellow arrowheads), with probes flanking the target lesion and satisfactory for ablation

Both single-probe and multiprobe (“chopstick”) configurations were employed, reflecting iterative refinement during this IDEAL stage 2a development phase. The “chopstick” technique was introduced once accurate single-probe placement had been established, allowing conformal ablation of larger or irregular lesions. Including both configurations enabled evaluation of robotic guidance across the full spectrum of procedural complexity encountered in early clinical practice (Figs. 2, 3).

### Cryoablation protocol

Ablation followed a triple-freeze protocol: 3 min freeze—3–4 min thaw—7 min freeze—3–5 min thaw—10 min freeze, followed by passive thaw until probe removal [12]. Ablation zone development was monitored by intermittent spiral CT. Track ablation was not performed. A post-ablation CT was performed to assess ground-glass opacity (GGO) as a surrogate marker for the ablation zone, evaluate complete coverage of the tumour and intended margin, and identify complications.

### Image analysis and follow-up

Targeting errors were measured for each probe by fusing the planning and CTs following the conclusion of the first probe placement on the robotic workstation. First cryoprobe placement Euclidean error (3D distance from the planned target to the needle tip) and lateral error (perpendicular deviation from the planned trajectory at target depth) were measured using orthogonal reformats aligned as closely as possible to the planned needle path (Fig. 3).

Participants underwent an initial post-ablation scan at 6 weeks to assess for residual tumour, followed by surveillance imaging at approximately 3 months and 6 months, and every 6 months thereafter. DICOM images were reviewed on a dedicated PACS workstation (IDS7, SECTRA) by an interventional radiologist (E.J.). Pulmonary transgression length (cryoprobe path through lung) was measured for each probe using multiplanar reformats.

All available multimodality follow-up imaging (CT and PET/CT) was reviewed to assess for residual unablated tumour and local tumour progression (LTP) at the ablation site [13]. Reports were generated by expert oncological radiologists or nuclear medicine physicians as part of routine clinical care.

### Data collection and outcome measures

Data were collected prospectively at four hierarchical levels—participant, procedure, lesion and probe using structured templates, electronic hospital records, procedural reports, and imaging review. Full variable definitions are available in Supplementary Table 1.

Primary outcomes included:

- Feasibility, defined as successful targeting of ≥ 90% of tumours using robotic guidance, without conversion to freehand technique (i.e., undocking the robot and proceeding with manual workflow).
- Safety, reported per intervention, based on procedural complication rate and severity (measured as per Common Terminology Criteria for Adverse Events (CTCAE v5.0) [14]). Events of interest included pneumothorax (timing judged on post-procedure CT and 2-h chest radiograph), chest drain insertion, and clinically significant haemorrhage.

Secondary outcomes included:

- Technical performance: planned trajectory metrics (orbital and craniocaudal angulation, target depth); targeting accuracy (Euclidean and lateral errors on first cryoprobe placement [15]); number of needle manipulations; targeting time (planning scan to completion of initial needle placement); total procedure time (planning scan to final scan); and radiation dose-length product for targeting and for the whole procedure.
- Technical success, defined as complete coverage of the target tumour by ground-glass opacification, judged by visual inspection [16].
- Technique evolution, including changes in probe selection, configuration and patient positioning strategy over time.

The exploratory outcomes were signals of primary and secondary technique efficacy, absence of residual unablated tumour or LTP using all available follow-up imaging.

All procedures were performed by two consultant interventional radiologists (E.J., N.F.) with extensive experience in robotic ablation (> 2 years and > 200 prior cases), obviating the need for formal learning curve analysis.

### Statistics

#### Study size

This prospective development study was aligned with IDEAL Stage 2a methodology, which typically involves cohorts of less than 30 participants [10]. Consequently, no formal sample size calculation was performed. Thus, 26 participants were enrolled pragmatically to facilitate iterative technical refinement, evaluate feasibility, and identify workflow or safety issues.

Continuous variables were assessed for normality using the Shapiro–Wilk test and summarised as mean ± standard deviation or median with interquartile range, as appropriate. Categorical variables were expressed as frequencies and percentages. Time to LTP was analysed using the Kaplan–Meier method, with censoring at the most recent imaging in participants without recurrence.

Analyses were performed using Microsoft Excel for Mac (version 16.98, Microsoft Corporation) and GraphPad Prism (v10.4.0, GraphPad Software).

## Results

### Baseline characteristics

In total, 26 participants (median age: 62 years, IQR 47–71; 14 men) underwent 30 robotic cryoablation procedure sessions targeting 37 tumours using 54 cryoprobes between June 2023 and June 2025. Most tumours were in the lower lobes (*n* = 20/37, 54%). Median tumour size was 9.8 mm (IQR: 5.1–12.8 mm), and the median distance from pleura was 0 mm (IQR: 0–7.2 mm) with 51% of tumours abutting the pleura and 89% lying within 10 mm. Most participants had colorectal cancer (*n* = 23, 89%). Detailed baseline characteristics are summarised in Table 1.

**Table 1:** Participant and tumour characteristics

Characteristic	Value
Participants	
Number of participants	26
Median age, years (IQR)	62 (47–71)
Sex, *n* (%)	Men: 14 (54%)
	Women: 12 (46%)
Charlson Comorbidity Score, median (IQR)	0 (0–0)
Primary tumour, *n* (%)	
Colorectal	23/26 (88%)
Sarcoma	2/26 (8%)
Lung	1/26 (4%)
Concurrent ablations, *n*	
Microwave liver ablation	2
Radiofrequency lung ablation	4
Number of procedures	30
Number of target tumours	37
Median lesion size, mm (IQR)	9.8 (5.1–12.8 mm)
Median distance from pleura, mm (IQR)	0 (0–7.2 mm)
Tumour location, *n* (%)	
Right upper lobe (RUL)	9/37 (24%)
Right middle lobe (RML)	2/37 (5%)
Right lower lobe (RLL)	10/37 (27%)
Left upper lobe (LUL)	6/37 (16%)
Left lower lobe (LLL)	10/37 (27%)

Values are presented as median (interquartile range) or number (%), unless otherwise stated

### Feasibility

Robotic guidance was successfully employed in 35/37 (95%) of procedures without conversion to freehand technique, meeting the pre-specified feasibility criterion of > 90%. In two cases, initial robotic insertion contacted a rib; conversion to freehand was judged more efficient than replanning, requiring undocking.

### Safety

One major complication (CTCAE v5.0 Grade 3) occurred—a pneumothorax requiring chest drain placement for 4 days with intermittent suction. All other events were Grade 1–2, with an overall complication rate (CTCAE Grade ≥ 1) of 67% (20/30), driven predominantly by pneumothoraces (*n* = 18), and including bleeding (*n* = 1), hypoxia (*n* = 1), fever (*n* = 1), pleural effusion (*n* = 1), and post-procedural pain (*n* = 3). Pneumothoraces were managed by observation (*n* = 7) or prophylactic intraprocedural chest drain insertion (*n* = 11, including the Grade 3 case), with 16/18 (89%) occurring after probe removal. No bronchopleural fistulas were encountered. All other events were grade 1 (low-grade fever, mild pain, pleural effusion). Median hospital stay was 1 night (IQR: 1–2). A detailed breakdown of procedural feasibility, complications, and post-procedural outcomes is provided in Table 2.

**Table 2:** Feasibility and safety outcomes

Outcome	Value	CTCAE grade
Tumour targeting completed with robotic guidance without conversion	35/37 (95%)	–
Any complication (CTCAE ≥ 1)	20/30 (67%)	–
Major complications (CTCAE ≥ 3)	1/30 (3%)	–
Pneumothorax	Total 18/30 (60%)	
	7/30	1 (observation)
	10/30 (37%)	2 (prophylactic drain insertion)
	1/30 (3%)	3 (prolonged prophylactic, requiring suction)
Timing of pneumothorax		
On insertion	2/18 (11%)	
After removal	16/18 (89%)	
Of which on delayed CXR	2/16	
Clinically apparent bleeding (clot requiring suction)	1/30 (3%)	2
Hypoxia requiring oxygen	1/30 (3%)	2
Fever	1/30 (3%)	1 (< 39 °C, < 24 h precautionary IV antibiotics, then oral)
Pleural effusion	1/30 (3%)	1
Post-procedural pain requiring analgesia	3/30 (10%)	1
Median post-procedure NPRS (IQR)	0 (0–0.5)	–
Bronchopleural fistula	0/30 (0%)	–
Unplanned CCU admission	0/30 (0%)	–
Air embolism/cryoshock/procedure-related death	0/30 (0%)	–
Hospital stay (nights), median (IQR)	1 (1–2)	–

Summary of procedural outcomes for all robotic lung cryoablation cases, including feasibility (conversion rate) and safety (complication rates and types). Values are presented as *n* (%), median (IQR or range) as appropriate

*CTCAE* common terminology criteria for adverse effects, *NPRS* numeric pain rating scale

### Technical performance

For the 54 cryoprobe insertions, targeting accuracy was high, with median first cryoprobe placement Euclidean error of 6.1 mm (IQR 2.9–9.7 mm) and lateral errors of 4.2 mm (IQR 2.2–6.5 mm). The median total procedure time (from initial planning scan to final CT) was 66.5 min (IQR 56.6–92.8). The median radiation dose-length product (DLP) was 1193 mGy·cm (IQR 775–1674). Detailed metrics of targeting accuracy, needle manipulation, trajectory complexity, duration, and radiation exposure are summarised in Table 3.

**Table 3:** Technical performance metrics of robot-assisted lung cryoablation procedures

Parameter	Median	IQR/range
Median number of cryoprobes per procedure	1	1–2
Use of chopstick technique, *n* (%)	17/37 (46%)	–
Targeting accuracy (first cryoprobe placement)		
Euclidean error, mm	6.1	2.9–9.7
Lateral error, mm	4.2	2.2–6.5
Needle manipulation		
Manipulations per needle	1	0–2.5
Placements requiring no adjustment, *n* (%)	17/35 (32%)	–
Trajectory complexity		
Orbital angulation, °	34.8	Range −45.7 to +89.1
Craniocaudal angulation, °	0.0	Range −18.1 to +43.4
Target depth, mm	86.7	Range 45.3 to 181.5
Path through lung, mm	50.8	Range 0.0 to 99.4
Procedure metrics		
Total procedure time, min	66.5	56.6–92.8
Targeting procedure time, min	21	10–30
Total radiation dose, DLP mGy·cm	1193	775–1674
Targeting radiation dose, DLP mGy·cm	528	315–662

For trajectory complexity parameters, values represent the range

*DLP* dose-length product

### Technical success

**Fig. 4:**
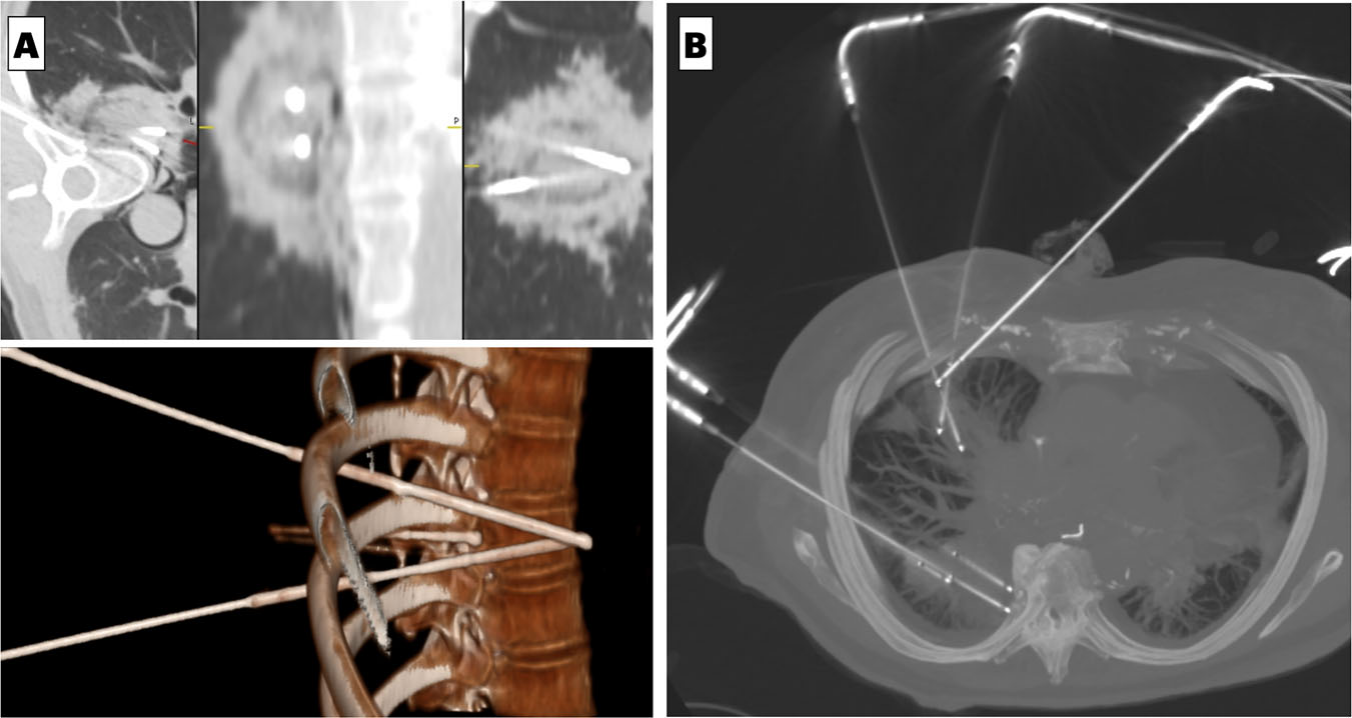
Examples of advanced robotic targeting strategies in complex lung cryoablation cases. **A** A 36-year-old woman undergoing cryoablation of a right lower-lobe colorectal metastasis. A paravertebral thermocouple was placed to monitor for potential ice propagation, as the planned ablation zone abutted the vertebral body, and ice extent cannot be visualised through bone on CT. This illustrates the integration of neuroprotection strategies into complex multiprobe targeting plans. **B** A 79-year-old woman undergoing cryoablation of four right lung colorectal cancer metastases using seven cryoprobes. The case demonstrates high procedural complexity and the capacity of robotic assistance to manage multitarget, multiprobe configurations

Technical success was achieved in 36/37 (97%) tumours. Examples of advanced targeting are illustrated in Fig. 4.

### Early efficacy

**Fig. 5:**
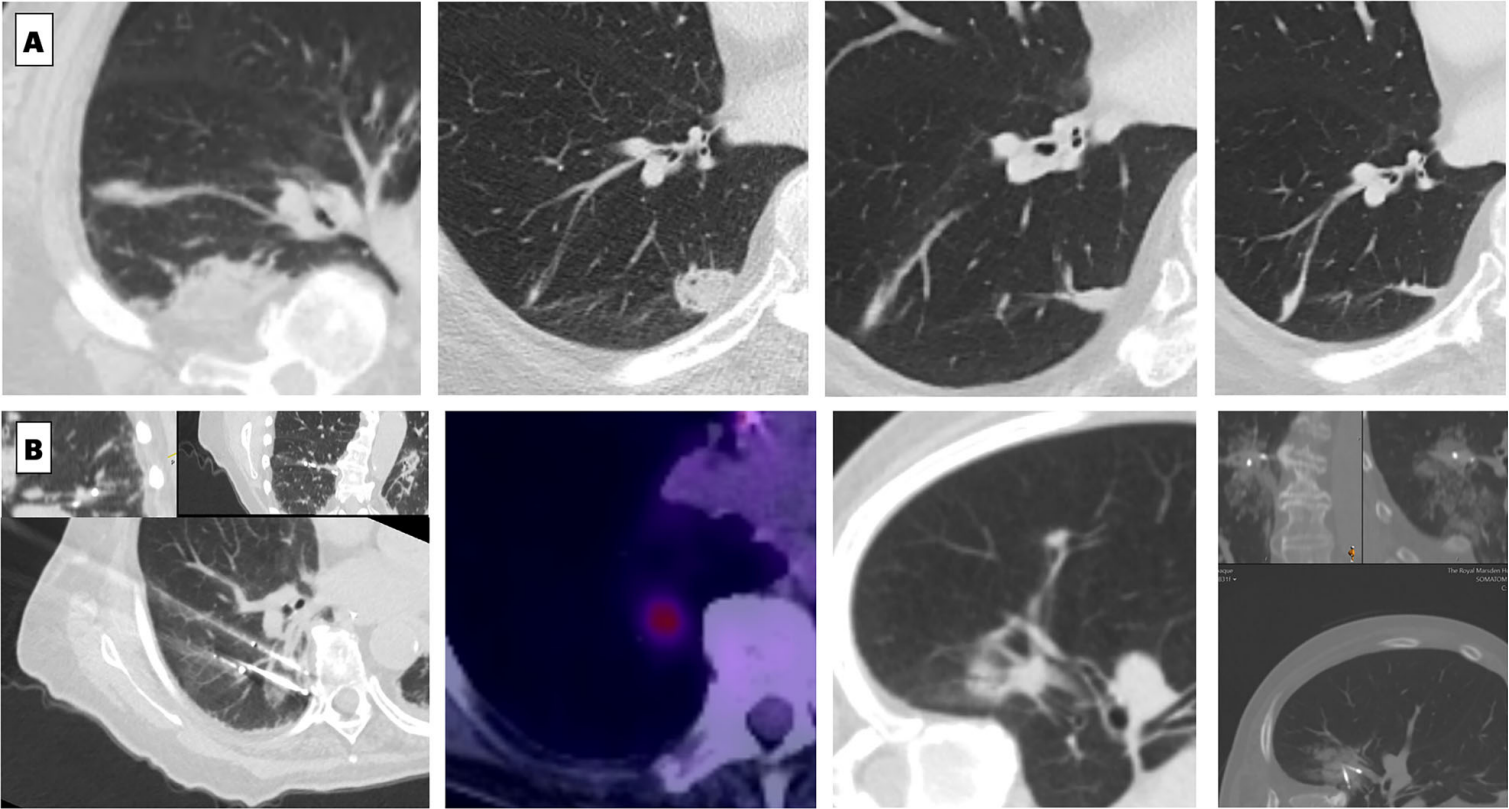
Examples of response to robot-assisted cryoablation. **A** Durable response to cryoablation. A 76-year-old man underwent ablation of a right lower-lobe rectal cancer metastasis. Serial CT images demonstrate involution of the ablation zone over time: immediately post-ablation, at 6 weeks, 4.5 months, and 11 months, without evidence of recurrence. **B** Local tumour progression following cryoablation. A 79-year-old woman with colorectal metastases underwent ablation of a right lower-lobe tumour. At the time, needle placement appeared satisfactory; however, in hindsight, the superior-inferior coverage was likely insufficient, which highlights the need to carefully review multiplanar reformats during needle placement. The FDG PET/CT 8.3 months post-ablation showed medial recurrence, subsequently treated by repeat cryoablation. Follow-up is ongoing

Median follow-up imaging for assessment of LTP was 193 days (IQR 169–397). The single case without technical success demonstrated residual unablated tumour on baseline imaging and was treated with radiotherapy. This was excluded from LTPFS analysis but counted as a failure for both primary and secondary technique efficacy. One LTP occurred at 8.3 months and was successfully managed with repeat cryoablation. Primary and secondary technique efficacy were 94% and 97%, respectively (Table 4). On Kaplan–Meier analysis (Supplementary Fig. 1), LTPFS was 100% at 6 months and 97.0% at 12 months. Examples of durable response and LTP are provided in Fig. 5.

**Table 4:** Early efficacy outcomes

Outcome	Value
Residual unablated tumour on baseline imaging	1/35 (2.9%)
Local tumour progression (LTP) on initial follow-up imaging	1/34 (2.9%)
Primary technique efficacy	33/35 (94.3%)
Secondary technique efficacy (after retreatment)	34/35 (97.1%)
6-month LTPFS	33/33 (100%)
12-month LTPFS	32/33 (97.0%)

*LTPFS* local tumor progression free survival

### Technique evolution

Probe selection and procedural workflow evolved during the study. We initially used 14 G (IceForce) probes for their broad ablation zones in the first five cases, then transitioned to smaller 17 G (IceRod or IceSphere) probes with increasing experience in single-needle planning. Configuration was based on target size, geometry, and location: single 17 G probes for tumours < 10 mm, and chopstick configurations for broader/conformal zones. After integration into our workflow, the chopstick technique was used in 17/31 tumours (55%), reflecting refined multiprobe planning and simultaneous insertion.

Patient positioning (ipsilateral-up vs. dependent) was selected according to lesion location and trajectory. Ipsilateral-down was favoured for higher bleeding-risk cases (Supplementary Fig. 2), as it may limit contralateral contamination and help reduce the rare but recognised risk of air embolism [17].

Simultaneous multiprobe insertion without interim scanning was used for selected peripheral lesions where incurred pneumothorax would not compromise safety—most commonly subpleural tumours without proximity to critical mediastinal or hilar structures. This change was informed by early observations that pneumothoraces occurred on probe removal.

Adaptations were intended to streamline the workflow while maintaining safety.

## Discussion

This prospective study is, to our knowledge, the first to evaluate robot-assisted CT-guided cryoablation for pulmonary metastases. We demonstrated feasibility and safety, with 95% successful robotic targeting and a low major complication rate (3.3%). Compared with our earlier robotic RFA work using simpler single-needle trajectories [9], the greater procedural complexity of multiprobe cryoablation appears to maximise the advantages of robotic planning and guidance. Subpleural metastases may be superficially located but are frequently constrained by rib proximity and pleural abutment, leaving little “run-up” to the target. Traversing a short segment of normal lung can help minimise the risk of bronchopleural fistula, yet this, combined with the need for precise multiprobe alignment, often requires oblique or double-oblique trajectories. These geometric constraints can make subpleural ablation technically demanding and highlight the advantages of robotic navigation for accurate probe placement.

Targeting accuracy was high (median Euclidean error 6.1 mm on first placement), comparable to our prior lung RFA experience (~5 mm) [9] though lower than for stereotactic liver ablation (meta-analysis: 3.7 mm) [7]. Even lower errors (1.6 mm) have been reported using a now-discontinued platform with greater autonomy and dynamic trajectory correction [18], although direct comparison is limited by differences in tissue characteristics, respiratory motion, and applicator properties. In our series, deviations were readily corrected with minimal additional manual manipulations (IQR 0–2.5).

Despite these minimal manipulations, to our surprise, pneumothorax rates were high (18/30, 60%), with intraprocedural drainage in 11/18 (61%; overall 37%). Most (89%) occurred after probe removal, suggesting cryoprobes tamponade bronchiolar disruptions until withdrawal unseals the tract, consistent with a lung biopsy study reporting dwell time does not influence pneumothorax risk [19]. Rates exceeded our previous robotic RFA series (30% pneumothorax, 10% drainage) [9] and other reports [20], likely reflecting the combined effect of multiprobe configurations and subpleural tumour location. No delayed drains occurred; only one remained > 24 h. Our low threshold for drainage may have prevented reintervention and contributed to short hospital stays (median 1 night), like thoracic surgery, where drains are routine. For example, SOLSTICE reported lower drain use (26%) but delayed drainage in 3%, and longer dwell times (> 3 days in 27.3%) [2]. Although manipulations may not drive pneumothorax, they still cause non-target trauma, obscure tumours via haemorrhage, and increase procedural complexity. This added cognitive and technical load may reduce efficiency, impair operator performance, and ultimately affect outcomes.

Our haemorrhage rate (3.3%) compares favourably with SOLSTICE and ECLIPSE (7.1% and 8.6%) [1, 2]. The only clinically apparent bleed followed placement of a single 14 G cryoprobe; up to seven 17 G probes were used in three lesions without bleeding. Distributing energy via multiple smaller probes may reduce focal trauma; ipsilateral-down positioning may protect the contralateral lung from blood spillage. These refinements, though developed in a robotic workflow, are equally applicable to freehand cryoablation.

The median total DLP in our series (1193 mGy·cm; ≈ 17 mSv) is consistent with values reported for contemporary CT-guided interventions. Recent robotic and navigated-CT studies have shown comparable or lower exposures (≈ 800–1600 mGy·cm; 11–22 mSv) [18, 21], while earlier helical cryoablation series reported much higher doses (≈ 7900 mGy·cm; 120 mSv) [22].

Limitations include the single-centre design, small sample size, and absence of a comparator arm. However, these are consistent with IDEAL Stage 2a development, in which iterative refinement is encouraged before progressing to randomised trials [10]. Although we did not formally compare pre- and post-robotic case selection, our experience strongly suggests the platform facilitates more technically challenging cases. As the Cardiovascular and Interventional Radiological Society of Europe (CIRSE) recommends 24-month follow-up [23], oncological outcomes remain preliminary, though early LTP rates are encouraging.

Although the robotic navigation platform is device-agnostic and could be used with radiofrequency or microwave ablation, we focused on cryoablation in this study. Cryoablation is especially advantageous for subpleural lesions because its collagen-sparing mechanism reduces the risk of bronchopleural fistula. Furthermore, it often requires multiprobe configurations and multiprobe spacing, which are difficult to achieve freehand but well-suited to robotic guidance.

Our cohort primarily comprised subpleural metastases. More central lesions are generally managed with radiofrequency ablation in our practice, which is faster and more cost-effective in that setting. The few central tumours treated were selected due to specific considerations, such as concomitant subpleural ablation within the same session or potential air embolism risk from bronchovenous fistula, where cryoablation may offer a safety advantage. Consequently, our findings may not be directly generalisable to central lesions, which remain therapeutically complex and warrant dedicated evaluation given their limited surgical and SABR options [24, 25].

Our findings support progression to a Stage 2b study, and ultimately Stage 3 IDEAL multicentre RCT comparing robotic and freehand cryoablation and assessing whether robotics extends the spectrum of lesions treatable with safety and efficacy.

## Conclusion

Robotic guidance offers particular advantages for cryoablation, enabling accurate and reproducible multiprobe configurations for conformal ice-ball formation with minimal manipulation. Using robotic CT guidance, we successfully performed cryoablation of subpleural pulmonary metastases, demonstrating feasibility, safety, and accuracy in technically complex cases. These findings suggest robotics may expand the treatable case spectrum and support a multicentre trial comparing robotic and freehand cryoablation.

## Supplementary information

Supplementary information

## Abbreviations

CTCAE: Common terminology criteria for adverse events
DLP: Dose-length product
IDEAL: Idea, Development, Exploration, Assessment, Long-term study
IQR: Interquartile range
LTP(FS): Local tumour progression (-free survival)

## References

[1] de Baère T, Woodrum D, Tselikas L et al (2021) The ECLIPSE study: efficacy of cryoablation on metastatic lung tumors with a 5-year follow-up. J Thorac Oncol 16:1840–1849. 10.1016/j.jtho.2021.07.021 34384914 10.1016/j.jtho.2021.07.021

[2] Callstrom MR, Woodrum DA, Nichols FC et al (2020) Multicenter study of metastatic lung tumors targeted by interventional cryoablation evaluation (SOLSTICE). J Thorac Oncol 15:1200–1209. 10.1016/j.jtho.2020.02.022 32151777 10.1016/j.jtho.2020.02.022 PMC9201766

[3] Abrishami Kashani M, Murphy MC, Saenger JA et al (2023) Risk of persistent air leaks following percutaneous cryoablation and microwave ablation of peripheral lung tumors. Eur Radiol 33:5740–5751 36892641 10.1007/s00330-023-09499-y

[4] Kawamura M, Izumi Y, Tsukada N et al (2006) Percutaneous cryoablation of small pulmonary malignant tumors under computed tomographic guidance with local anesthesia for nonsurgical candidates. J Thorac Cardiovasc Surg 131:1007–1013 16678583 10.1016/j.jtcvs.2005.12.051

[5] Inoue M, Nakatsuka S, Yashiro H et al (2012) Percutaneous cryoablation of lung tumors: feasibility and safety. J Vasc Interv Radiol 23:295–302 22265246 10.1016/j.jvir.2011.11.019

[6] Rivera AKU, Seeliger B, Goffin L, Garcia-Vazquez A, Mutter D, Gimenez ME (2024) Robotic assistance in percutaneous liver ablation therapies: a systematic review and meta-analysis. Ann Surg Open 5:e406 38911657 10.1097/AS9.0000000000000406 PMC11191991

[7] Tinguely P, Paolucci I, Ruiter SJS et al (2021) Stereotactic and robotic minimally invasive thermal ablation of malignant liver tumors: a systematic review and meta-analysis. Front Oncol. 10.3389/fonc.2021.713685 10.3389/fonc.2021.713685 PMC8495244 34631539

[8] Beermann M, Lindeberg J, Engstrand J et al (2019) 1000 consecutive ablation sessions in the era of computer assisted image guidance—lessons learned. Eur J Radiol Open 6:1–8. 10.1016/j.ejro.2018.11.002 30547062 10.1016/j.ejro.2018.11.002 PMC6282637

[9] Johnston EW, Basso J, Silva F et al (2023) Robotic versus freehand CT-guided radiofrequency ablation of pulmonary metastases: a comparative cohort study. Int J Comput Assist Radiol Surg. 10.1007/s11548-023-02895-1 10.1007/s11548-023-02895-1 PMC10497639 37072657

[10] McCulloch P, Altman DG, Campbell WB et al (2009) No surgical innovation without evaluation: the IDEAL recommendations. Lancet 374:1105–1112 19782876 10.1016/S0140-6736(09)61116-8

[11] Johnston EW, Basso J, Winfield J et al (2022) Starting CT guided robotic interventional oncology at a UK centre. Br J Radiol. 10.1259/bjr.20220217 10.1259/bjr.20220217 PMC10996412 35290098

[12] Hinshaw JL, Littrup PJ, Durick N et al (2010) Optimizing the protocol for pulmonary cryoablation: a comparison of a dual-and triple-freeze protocol. Cardiovasc Interv Radiol 33:1180–1185 10.1007/s00270-010-9868-0 PMC3155994 20437048

[13] Ahmed M, Solbiati L, Brace CL et al (2014) Image-guided tumor ablation: standardization of terminology and reporting criteria—a 10-year update. Radiology 273:241–260 24927329 10.1148/radiol.14132958 PMC4263618

[14] National Cancer Institute (2017) Common terminology criteria for adverse events (CTCAE) version 5.0. https://dctd.cancer.gov/research/ctep-trials/for-sites/adverse-events

[15] Spenkelink IM, Heidkamp J, Avital Y, Fütterer JJ (2023) Evaluation of the performance of robot assisted CT-guided percutaneous needle insertion: comparison with freehand insertion in a phantom. Eur J Radiol 162:110753 36863276 10.1016/j.ejrad.2023.110753

[16] De Baere T, Tselikas L, Woodrum D et al (2015) Evaluating cryoablation of metastatic lung tumors in patients—safety and efficacy the ECLIPSE trial—interim analysis at 1 year. J Thorac Oncol 10:1468–1474 26230972 10.1097/JTO.0000000000000632

[17] Glodny B, Schönherr E, Freund MC et al (2017) Measures to prevent air embolism in transthoracic biopsy of the lung. AJR Am J Roentgenol 208:W184–W191 28301208 10.2214/AJR.16.16048

[18] Levy S, Goldberg SN, Roth I et al (2021) Clinical evaluation of a robotic system for precise CT-guided percutaneous procedures. Abdom Radiol (NY) 46:5007–5016. 10.1007/s00261-021-03175-9 10.1007/s00261-021-03175-9 34146132

[19] Ko JP, Shepard J-AO, Drucker EA et al (2001) Factors influencing pneumothorax rate at lung biopsy: are dwell time and angle of pleural puncture contributing factors? Radiology 218:491–496 11161167 10.1148/radiology.218.2.r01fe33491

[20] Eiken PW, Welch BT (2019) Cryoablation of lung metastases: review of recent literature and ablation technique. Semin Interv Radiol 36:319–325 10.1055/s-0039-1697002 PMC6823100 31680723

[21] Yang K, Ganguli S, DeLorenzo MC, Zheng H, li X, Liu B (2018) Procedure-specific CT dose and utilization factors for CT-guided interventional procedures. Radiology 289:150–157 30015583 10.1148/radiol.2018172945

[22] Leng S, Christner JA, Carlson SK et al (2011) Radiation dose levels for interventional CT procedures. AJR Am J Roentgenol 197:97–103. 10.2214/AJR.10.5057 21701002 10.2214/AJR.10.5057

[23] Venturini M, Cariati M, Marra P, Masala S, Pereira PL, Carrafiello G (2020) CIRSE standards of practice on thermal ablation of primary and secondary lung tumours. Cardiovasc Interv Radiol 43:667–683. 10.1007/s00270-020-02432-6 10.1007/s00270-020-02432-6 32095842

[24] UK SABR Consortium (2019) Stereotactic ablative body radiation therapy (SABR): a resource. Version 6.1. Available via https://www.sabr.org.uk/wp-content/uploads/2019/04/SABRconsortium-guidelines-2019-v6.1.0.pdf. Accessed 25 Oct 2025

[25] Bai G, Chen X, Peng Y et al (2024) Surgery challenges and postoperative complications of lung cancer after neoadjuvant immunotherapy. Thorac Cancer 15:1138–1148 38572774 10.1111/1759-7714.15297 PMC11091790

