# Supplementary material for: Robot-assisted CT-guided cryoablation of pulmonary metastases: an IDEAL stage 2a prospective development study
Source: Eur Radiol. 2026 Feb 5;36(7):5857–67. doi: 10.1007/s00330-026-12335-8 (PMC13282352; doi:10.1007/s00330-026-12335-8)
Supplement: Supplementary file 1 — Supplementary information [file 330_2026_12335_MOESM1_ESM.pdf]

Robot-Assisted CT-Guided Cryoablation of Pulmonary Metastases: An IDEAL Stage 2a Prospective Development Study

ELECTRONIC SUPPLEMENTARY MATERIAL

Supplementary Table 1. Data Dictionary

Level	Variable	Definition / Notes
Participant	Age	At time of first procedure
	Sex	Male / Female
	Primary tumour type	e.g. colorectal, lung etc.
	Charlson Comorbidity Index	Calculated per Charlson et al. [1]
Procedure	Number of target tumours	Treated during single procedure
	Total procedure time	From planning CT to final CT (excluding anaesthesia setup)
	Targeting time	From planning CT to final cryoprobe positioning
	Total procedure radiation dose	CT dose-length product (DLP, mGy·cm) of all scans
	Targeting radiation dose	CT dose-length product (DLP, mGy·cm) from planning CT to final cryoprobe positioning
	Complications	Graded using CTCAE version 5
	Pneumothorax	Confirmed on CT or chest radiograph<br>Timing related to probe insertion/removal
	Chest drain insertion	Yes/No
	Hospital admission duration	Nights
Tumour	Lobe / side	Anatomical location
	Tumour size	Longest axial dimension (mm)
	Distance from pleura	Measured on axial images
	Number and type of probes	IceRod / IceSphere / IceForce
	Ablation adequacy	GGO coverage on post-ablation CT
	Trajectory complexity	Orbital angulation°, cranio-caudal angulation, chopsticks technique
	Transpulmonary path length (mm)	Length of cryoprobe in lung in final treatment position
Probe	Euclidean error	3D distance from planned to actual tip position (mm)
	Lateral error	Perpendicular distance from planned trajectory to needle shaft at target depth (mm)
	Needle manipulations	Any repositioning or redirection
	Probe depth	Distance from pleural entry to lesion centre (mm)

GGO; ground glass opacification.

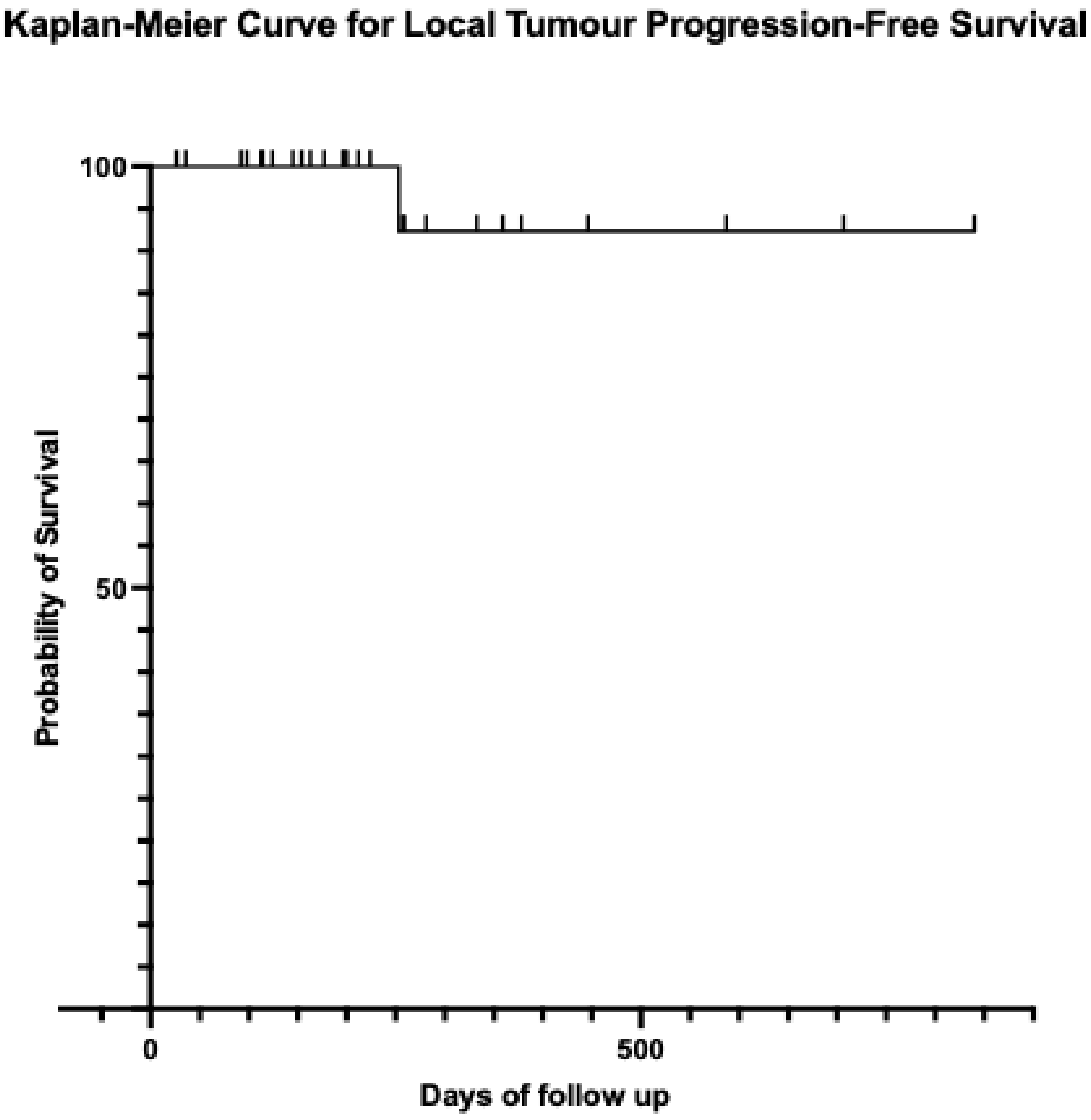

Supplementary Figure 1. Kaplan–Meier curve showing local tumour progression-free survival (LTPFS) following robotic-assisted cryoablation of metastatic lung tumours.

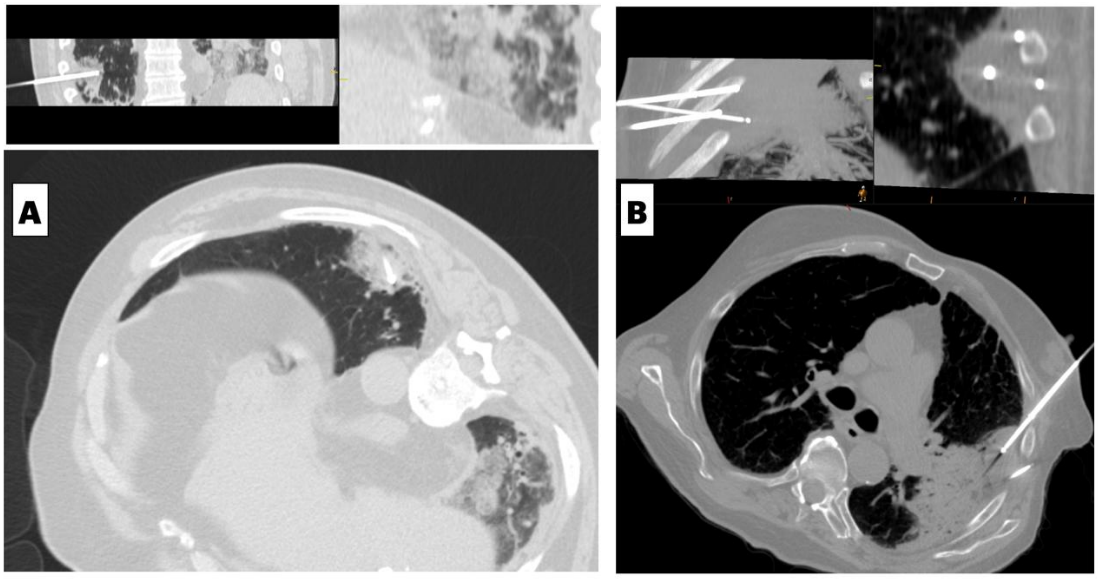

Supplementary Figure 2. Illustrative examples highlighting the importance of patient positioning during cryoablation.

A. A 78-year-old man undergoing cryoablation for a subpleural left lower lobe colorectal metastasis using a single 14G cryoprobe. Positioning with the left (cryoprobe) side up allowed blood to accumulate in the major airways and track into the dependent lung, resulting in contralateral contamination (visible as ground glass opacification).

B. A 77-year-old man (case outside the study cohort, shown for illustrative purposes) undergoing cryoablation of a left lower lobe lung cancer metastasis using three 17G cryoprobes. Despite moderate haemorrhage, deliberate left lateral oblique decubitus positioning preserved the contralateral lung and prevented spillage of blood or fluid into the right bronchial tree, and did not cause any clinical sequelae.

References

1. Charlson ME, Pompei P, Ales KL, MacKenzie CR. A new method of classifying prognostic comorbidity in longitudinal studies: development and validation. J Chronic Dis. 1987;40(5):373–83.
